# Supplementary material for: Innate Immune Response Analysis in Meniscus Xenotransplantation Using Normal and Triple Knockout Jeju Native Pigs
Source: Int J Mol Sci. 2022 Sep 8;23(18):10416. doi: 10.3390/ijms231810416 (PMC9499368; doi:10.3390/ijms231810416)
Supplement: Supplementary file 1 [file ijms-23-10416-s001.zip › ijms-1894962-supplementary.pdf]

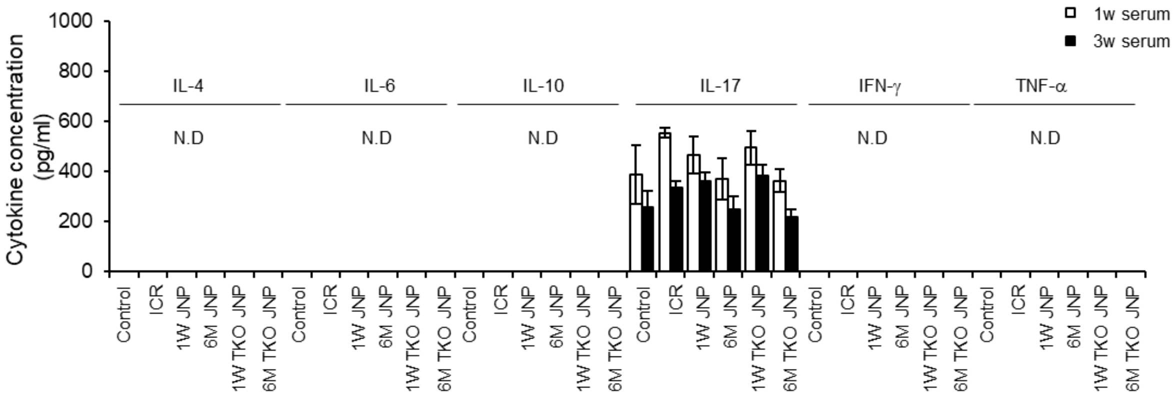

Figure S1. Cytokine concentrations of mouse blood in xenograft normal or TKO JNP menisci in mice. Blood was collected at 1 week and 3 weeks during experiment periods. The cytokine levels of IL-4, IL-6, IL-10, IL-17, IFN-γ, and TNF-α were analyzed for the systemic immune response.
